# Supplementary material for: Aggressive strategies of the COVID-19 pandemic on the apparel industry of Sri Lanka using structural equation modeling
Source: PLoS One. 2023 Jun 21;18(6):e0286717. doi: 10.1371/journal.pone.0286717 (PMC10284376; doi:10.1371/journal.pone.0286717)
Supplement: Attachment — Submitted filename: Responses to Reviewers.docx [file pone.0286717.s001.docx]

| **Date:** | Mar 14 2023 |
| --- | --- |
| **To:** | "PLOS ONE" plosone@plos.org |
| **From:** | "Wasantha Rajapakshe" wasantha.r@sliit.lk |
| **Subject:** | PLOS ONE Decision: Revision required [PONE-D-23-03480] |

Point by point response to editor and reviewers

Dear editor and the reviewers,

We would like to express our profound appreciation to the editor and the reviewers for the valuable comments and suggestions made on our manuscript which were very helpful in revising and improving it.

Please note that the line numbers referred to in this document is aligned with the file labeled 'Manuscript'.

**Journal requirements:**

When submitting your revision, we need you to address these additional requirements.

1. Please ensure that your manuscript meets PLOS ONE's style requirements, including those for file naming. The PLOS ONE style templates can be found at

https://journals.plos.org/plosone/s/file?id=wjVg/PLOSOne_formatting_sample_main_body.pdf and

<https://journals.plos.org/plosone/s/file?id=ba62/PLOSOne_formatting_sample_title_authors_affiliations.pdf>

**Comments of Authors:** This has been corrected in the revised version.

2. We suggest you thoroughly copyedit your manuscript for language usage, spelling, and grammar. If you do not know anyone who can help you do this, you may wish to consider employing a professional scientific editing service.

**Comments of Authors:** This has been corrected in the revised version. The revised version has been proofread. Corrections have been made including typos, comma correction and brevity.

Whilst you may use any professional scientific editing service of your choice, PLOS has partnered with both American Journal Experts (AJE) and Editage to provide discounted services to PLOS authors. Both organizations have experience helping authors meet PLOS guidelines and can provide language editing, translation, manuscript formatting, and figure formatting to ensure your manuscript meets our submission guidelines. To take advantage of our partnership with AJE, visit the AJE website (http://learn.aje.com/plos/) for a 15% discount off AJE services. To take advantage of our partnership with Editage, visit the Editage website (www.editage.com) and enter referral code PLOSEDIT for a 15% discount off Editage services.  If the PLOS editorial team finds any language issues in text that either AJE or Editage has edited, the service provider will re-edit the text for free.

Upon resubmission, please provide the following:

The name of the colleague or the details of the professional service that edited your manuscript

A copy of your manuscript showing your changes by either highlighting them or using track changes (uploaded as a *supporting information* file)

A clean copy of the edited manuscript (uploaded as the new *manuscript* file)

3. We note that you have indicated that data from this study are available upon request. PLOS only allows data to be available upon request if there are legal or ethical restrictions on sharing data publicly. For more information on unacceptable data access restrictions, please see http://journals.plos.org/plosone/s/data-availability#loc-unacceptable-data-access-restrictions.

In your revised cover letter, please address the following prompts:

a) If there are ethical or legal restrictions on sharing a de-identified data set, please explain them in detail (e.g., data contain potentially sensitive information, data are owned by a third-party organization, etc.) and who has imposed them (e.g., an ethics committee). Please also provide contact information for a data access committee, ethics committee, or other institutional body to which data requests may be sent.

b) If there are no restrictions, please upload the minimal anonymized data set necessary to replicate your study findings as either Supporting Information files or to a stable, public repository and provide us with the relevant URLs, DOIs, or accession numbers. For a list of acceptable repositories, please see http://journals.plos.org/plosone/s/data-availability#loc-recommended-repositories.

We will update your Data Availability statement on your behalf to reflect the information you provide.

Additional Editor Comments: You should carefully read through the comments and address the observations. I would like to suggest modifying the title and write clearly the novelty of the research.

**Comments of Authors:**

Noted with thank you. Title has been modified and novelty has been incorporated in the revised manuscript with track changes.

**Reviewers' comments:
Reviewer's Responses to Questions**

**Comments to the Author**

1. Is the manuscript technically sound, and do the data support the conclusions?

The manuscript must describe a technically sound piece of scientific research with data that supports the conclusions. Experiments must have been conducted rigorously, with appropriate controls, replication, and sample sizes. The conclusions must be drawn appropriately based on the data presented.

Reviewer #1: Partly

Reviewer #2: No

Reviewer #3: Partly

**Comments of Authors:** Noted with thank you. This has been incorporated in the revised manuscript with track changes. (Line 496-501)

*“The results found that majority of non-managerial (73.7%) employees are female (51.3%), with 21-30 years range (38.7%) have higher tendency to stress during pandemic situation. Most of them are in the 20,000-50,000-salary range (45.1%) with 5-9 years of service (35.2%) in the apparel sector. In conclusion, it notes that aggressive cost reduction, as well as environmental changes, have a direct impact on employee stress rather than on business sustainability. To reduce employee stress, companies must eliminate factors that directly influence stress. As per the findings, eliminating cost reduction and environmental changes has a direct impact on eliminating employees’ stress. Managers must focus on proposing policies for stress reduction”.*

 2. Has the statistical analysis been performed appropriately and rigorously?

 Reviewer #1: Yes

Reviewer #2: Yes

Reviewer #3: Yes

**Comments of Authors:** Well, noted. Thank you for your comment.

 3. Have the authors made all data underlying the findings in their manuscript fully available?

The [PLOS Data policy](http://www.plosone.org/static/policies.action#sharing) requires authors to make all data underlying the findings described in their manuscript fully available without restriction, with rare exception (please refer to the Data Availability Statement in the manuscript PDF file). The data should be provided as part of the manuscript or its supporting information, or deposited to a public repository. For example, in addition to summary statistics, the data points behind means, medians and variance measures should be available. If there are restrictions on publicly sharing data—e.g. participant privacy or use of data from a third party—those must be specified.

 Reviewer #1: No

Reviewer #2: Yes

Reviewer #3: Yes

**Comments of Authors:**

Thank you comment has been noted. Data cannot be shared publicly because of data protection. Data are available from the Sri Lanka Institute of Information Technology, SLIIT Business School, Sri Lanka (contact via [sandalimihara@gmail.com](mailto:sandalimihara@gmail.com)  or [wasantha.r@sliit.lk](mailto:wasantha.r@sliit.lk) ) for researchers who meet the criteria for access to confidential data. However, for the review’s purposes, we are sending the data file as a supporting document.

 4. Is the manuscript presented in an intelligible fashion and written in standard English?

PLOS ONE does not copyedit accepted manuscripts, so the language in submitted articles must be clear, correct, and unambiguous. Any typographical or grammatical errors should be corrected at revision, so please note any specific errors here.

Reviewer #1: Yes

Reviewer #2: Yes

Reviewer #3: No

**Comments of Authors:**

Thank you and well noted. This has been in cooperated in the revised manuscript with track changes.

 5. Review Comments to the Author

Please use the space provided to explain your answers to the questions above. You may also include additional comments for the author, including concerns about dual publication, research ethics, or publication ethics. (Please upload your review as an attachment if it exceeds 20,000 characters)

**Reviewer #1**

**Reviewer #1**: During the study author tested the hypotheses on 384 respondents by using the structured equation modeling with the aid of AMOS and concluded that aggressive cost reduction and environmental changes, have a direct impact on employee stress rather than on business sustainability.

Please revise the title of the paper. Usually, it is not suggested to use the question in titles. Because the aim of conducting research is to find answers and having a question in your title may not be attractive to the readers.

**Comments of Authors:** Thank you and well noted. Title has changed to

*“Aggressive Strategies of the COVID-19 Pandemic on the Apparel Industry of Sri Lanka Using Structural Equation Modeling”*

Please revise the abstract and include the result of empirical findings in the abstract.

**Comments of Authors:** Comment has been noted and this has been corrected in the revised manuscript. (Line 31-35)
*“Aggressive cost reduction strategies (Beta = 1.274, p = 0.000) and environmental changes (Beta = 0.251, p = 0.000) led to an increase in employee stress but did not affect on business sustainability. Thus, employees stress (Beta = -0.028, p = 0.594) was not a mediator in the relationship between aggressive cost cutting strategies and business sustainability and business sustainability was not a dependent variable.”*

Line 56 I cannot cross-check the references. In addition, cited statistics are old, and please cite the up-to-date statistic for 2022.

**Comments of Authors:**

Comment has been noted and this has been corrected in the revised manuscript in Line 69-71 with latest (2022) statistics with a new citation.

*“A study on waste generation in the garment industry in Sri Lanka proves that a considerable 39.4 % of the total wastes, followed by fabric and yarn is around 29.6% [10]”.*

Line 63, please revise the sentence “As countries…”

**Comments of Authors:** Comment has been noted and this has been corrected in the revised manuscript (Line 78-80).

*“International trade declined, supply chain operations were interrupted, and orders were canceled due to rapidly declining demand, showing the vulnerability of global supply chains to the COVID-19 pandemic situation.”.*

Line 67, Please specify earlier crises, such as....

**Comments of Authors:** Comment has been noted and this has been corrected in the revised manuscript (Line 81-82).

*“such as severe acute respiratory syndrome coronavirus (SARS-CoV) and Middle East respiratory syndrome coronavirus (MERS-CoV)”,*

Line 90, Please revise the paragraphs into the past tense as the pandemic is already over, and the world is moving forward. For instance, The Joint Apparel Association 90 Forum (JAAF) in Sri Lanka anticipated the epidemic.

**Comments of Authors:** Comment has been noted and this has been corrected in the revised manuscript (Line 108-110).

*“The Joint Apparel Association Forum (JAAF) in Sri Lanka anticipated that the pandemic affected 100,000 job losses in the apparel sector, with a 40% decrease in clothing and textile exports in the fiscal year 2020 [21]”.*

Line 124- One of the study purposes is to identify the impact of cost-cutting strategies on apparel strategies during Covid. While in the introduction, I can not see any information about the cost-cutting strategies what tactics or strategies had been adopted by the company to reduce the cost, while I think all the implementation, such as health and safety measures and completion of orders during Covid also increased the price due to high carriage rates.

**Comments of Authors:** Thank you for your comment. This has been corrected in the revised manuscript (Page 4 - line 139-142).

*“Many firms in sectors afflicted by the Covid-19 pandemic are deciding on salary cuts rather than layoffs to preserve their employees for a quick recovery [21]. Waste management, freezing recruitment and applying lean six sigma were basic tactics used by the firms to manage expenditure [7,11,19]. “*

I think the author should move the first paragraph of the literature into the introduction section, Lines 132-140, or explain the business suitability in the introduction section as well.

**Comments of Authors:** Thank you for your comment. This has been corrected in the revised manuscript (Page 2 - line 44-52 and page 4 – Line 164-167).

*“The management and coordination of environmental, social, and financial demands and concerns to achieve responsible, ethical, and long-term success are known as business sustainability [1]. Sustainable business strategies bring significant financial and environmental benefits to manufacturers, such as cost-effective procedures that reduce negative environmental consequences while preserving energy and natural resources. Employees, community, and product safety too are improved by sustainable production [2]. Three key components can be identified regarding business sustainability: employee satisfaction (people), long-term sustainable profit (profit), and a more sustainable planet with fewer resources polluting the environment (planet) [3].”*

*Line 164-167*

*“Sustainable manufacturing is defined as the production of manufactured goods using cost-effective procedures that reduce negative environmental consequences while preserving energy and natural resources. Employees, community, and product safety are all improved by sustainable production [2].”*

In line 173- Just in Time delivery Strategy, I wonder that in the introduction section author stated that due to the breakage in the supply chain and working from home decreased production, and most of the operations were shut down during the pandemic. How in-time delivery would reduce the carriage cost was high worldwide due to breakage in the supply chain.

**Comments of Authors:** Thank you for your comment and well noted. Even though there was interruption of supply chain, many researchers revealed that JIT was one way to apply warehouse cost reduction. Thus, with the literature support we think that also the possible strategy to reduce costs. However, we have adjusted the section as you mentioned.

I think the author should move lines 319 – 325 into the methodology section.

**Comments of Authors:** Thank you for your comment. We have corrected this in the manuscript (Line 273-279).

*“The SEM is also a widely used statistical modeling tool that can be used to integrate factor analysis and regression path analysis. This way, SEM helps researchers examine complex relationships based on a structural model by representing the hypothesis between the dependent and independent variables. According to [69], the SEM aims to expand the relationship between main variables and is surrounded by two primary models: the measurement model (basically confirmatory factor analysis) and the SEM.”*

Line 414: Frlimitationssult, the author concluded that “many apparel companies have moved high-quality automated systems because sometimes they have to cover two employee workloads with one employee due to increased absenteeism and how it is the cost reduction strategy in the short term if companies are investing huge amount on the machinery.
Please justify why the author adopted Structural equation modeling (SEM)

**Comments of Authors:** Thank you for your comment. (Line 273-279)

*“The SEM is also a widely used statistical modeling tool that can be used to integrate factor analysis and regression path analysis. This way, SEM helps researchers examine complex relationships based on a structural model by representing the hypothesis between the dependent and independent variables. According to [69], the SEM aims to expand the relationship between main variables and is surrounded by two primary models: the measurement model (basically confirmatory factor analysis) and the SEM.”*

Include the limitations and guidelines for further studies.

**Comments of Authors:** Thank you for your comment. We have corrected this in the manuscript. (Line 518-530)

“The issue of aggressive cost cutting while maintaining business sustainability is relatively new. As such, currently, a limited number of studies are available with gaps in the literature. This study contributes to this gap and focused on the Sri Lankan apparel industry, which is vital for apparel manufacturers to understand ways to reduce aggressive costs and control workplace environment changes, prioritizing workplace health and safety while maintaining business sustainability. The data were only limited to employees in the top 10 textiles and apparel manufacturing companies in Sri Lanka, and the sample size was 100 employees. The study's limitations include not promptly addressing issues of other severely impacted industries, such as tourism, education, transportation etc., and only reflecting the Sri Lankan apparel manufacturers' perspective in a situation where apparel manufacturers worldwide have been impacted. The authors recommend further research to investigate the COVID-19 impact on business sustainability, by considering small and medium-sized apparel companies in Sri Lanka.”

**Reviewer #2**

**Reviewer #2:** This study highlights the importance of investing in business sustainability, particularly in the apparel industry, in order to ensure survival in challenging situations such as the COVID-19 pandemic. The author notes that while there have been numerous studies on business sustainability, there is still a need for further investigation into how factors such as aggressive cost reduction, organizational environment change, and employee stress impact business sustainability.

The study conducted by the author involved 384 respondents (How this been determined, please show detail sample size calculation) in the Sri Lankan apparel industry and utilized structured equation modeling with the aid of AMOS to analyze the data. The key findings indicated that aggressive cost reduction and environmental changes have a direct impact on employee stress, rather than on business sustainability. The author suggests that policymakers should identify the real causes for business sustainability in order to minimize negative influences that could be affected by sustainability.

Overall, this study provides valuable insight into the importance of business sustainability in the apparel industry, particularly in the current pandemic situation, and highlights the need for further research in this area. The study's findings suggest that policymakers should take a comprehensive approach to business sustainability that takes into account a range of factors, including cost reduction, organizational environment change, and employee stress.

Provide sritics of the literature you reviewed. Also it it better to provide summary of the literature review and it needs to be reflected in the research gap or need of the study.

It is better to provide mean age (SD), in salary and year in service please show median ( Q1, Q3)

**Comments of Authors:** Thank you for your comment. We have corrected this in the manuscript (Line 307-317).

“Their average (standard deviation) age was 26.8 (SD =9.73) years.

Moreover, 101 out of 384 were at the managerial level, and 283 were at the non-managerial level; thus, 73.7% of employees in the sample were in the non-managerial group.

Meanwhile, 45.1% of employees earned a salary between Sri Lankan Rupees (Rs) 25,000 and 50,000 per month. Their Median Salary Level Q1 was Rs.25,000- 50,000 while in Q3, it was Rs.50,000- 70,000. Most employees (35.2%) have had working experience of between 5 to 9 years. Only 19 employees have worked for more than 25 years. In addition to that, 51 employees were with less than one year of experience. The descriptive statistics show the Median year in service Q1 was 5–9 years while Q3 was 1-4 years.”

Revise Intext citation formatting.

**Comments of Authors:** Thank you for your comment. We have corrected this in the manuscript.

Revise referencing formatting as per manuscript guideline again.

**Comments of Authors:** Thank you for your comment. We have corrected this in the manuscript.

**Reviewer #3**

**Reviewer #3:** 1- the aim of study did not mentioned clearly in the abstract, please rewrite the abstract and mention the purpose of study, method, findings & conclusion briefly.

**Comments of Authors:** Thank you for your comment. We have corrected this in the manuscript. (Line 21-39)

“During the COVID-19 crisis, the apparel industry faced many challenges. Aggressive cost cutting strategies became a top priority, and in turn, these influenced stressors and adversely affected business sustainability. This study examines the impact of aggressive strategies of the COVID-19 pandemic on business sustainability in the apparel industry of Sri Lanka. Further, it investigates whether the relationship between aggressive cost cutting strategies and business sustainability was mediated by employee stress considering aggressive cost reduction strategies and workplace environmental changes. This was a cross sectional study with data collected from 384 employees in the apparel industry in Sri Lanka. Structural Equation Modelling (SEM) modeling was applied to analyze the direct and indirect effects of aggressive cost reduction strategies and workplace environmental changes on sustainability with mediating effects of stress. Aggressive cost reduction strategies (Beta = 1.274, p = 0.000) and environmental changes (Beta = 0.251, p = 0.000) led to an increase in employee stress but did not affect on business sustainability. Thus, employees stress (Beta = -0.028, p = 0.594) was not a mediator in the relationship between aggressive cost cutting strategies and business sustainability and business sustainability was not a dependent variable. The findings proved that managing workplace stress, particularly improving stressful working environments and aggressive cost reduction strategies can enhance employee satisfaction. Thus, it is plausible that managing employee stress could be benefit for policy makers to focus on which area they should identify to retain competent employees. “

2- key words should be more relevant and correct based on MESH & COVID 19 should be added.

**Comments of Authors:** Thank you for your comment. We have corrected this in the manuscript. (Line 40-41)

*Key Words: Business Sustainability, Aggressive Strategies, Employee Stress, Apparel Industry, COVID-19, Sri Lanka*

3- many sentences ( e.g. first and second sentences of second paragraph) do not have any references, please add

**Comments of Authors:** Thank you for your comment. We have corrected this in the manuscript highlighted in red and with tract changes.

6. PLOS authors have the option to publish the peer review history of their article ([what does this mean?](https://journals.plos.org/plosone/s/editorial-and-peer-review-process#loc-peer-review-history)). If published, this will include your full peer review and any attached files.

**Comments of Authors:** Yes

If you choose “no”, your identity will remain anonymous but your review may still be made public.

**Do you want your identity to be public for this peer review?** For information about this choice, including consent withdrawal, please see our [Privacy Policy](https://www.plos.org/privacy-policy).

Reviewer #1: No

Reviewer #2: **Yes:** BIBHAV ADHIKARI

Reviewer #3: No

[NOTE: If reviewer comments were submitted as an attachment file, they will be attached to this email and accessible via the submission site. Please log into your account, locate the manuscript record, and check for the action link "View Attachments". If this link does not appear, there are no attachment files.]

While revising your submission, please upload your figure files to the Preflight Analysis and Conversion Engine (PACE) digital diagnostic tool, <https://pacev2.apexcovantage.com/>. PACE helps ensure that figures meet PLOS requirements. To use PACE, you must first register as a user. Registration is free. Then, login and navigate to the UPLOAD tab, where you will find detailed instructions on how to use the tool. If you encounter any issues or have any questions when using PACE, please email PLOS at [figures@plos.org](mailto:figures@plos.org). Please note that Supporting Information files do not need this step.

*In compliance with data protection regulations, you may request that we remove your personal registration details at any time.* [*(Remove my information/details)*](https://www.editorialmanager.com/pone/login.asp?a=r)*. Please contact the publication office if you have any questions.*
