# Supplementary material for: Aggressive strategies of the COVID-19 pandemic on the apparel industry of Sri Lanka using structural equation modeling
Source: PLoS One. 2023 Jun 21;18(6):e0286717. doi: 10.1371/journal.pone.0286717 (PMC10284376; doi:10.1371/journal.pone.0286717)
Supplement: Attachment — Submitted filename: Responses to Reviewers.docx [file pone.0286717.s002.docx]

**Date:** April 26, 2023

**To:** Prof. Yasir Ahmad
Academic Editor
PLOS ONE

**From:** Wasantha Rajapakshe

**Subject:** PONE-D-23-03480R1

Aggressive Strategies of the COVID-19 Pandemic on the Apparel Industry of Sri Lanka Using Structural Equation Modeling

**Point by point response to editor and reviewers**

Dear Respected Academic Editor and Reviewers,

We would like to express our profound appreciation to you for the valuable comments and suggestions made on our manuscript which were very helpful in revising and improving it.

Please note that the line numbers referred to in this document is aligned with the file labeled 'Manuscript'.

| **Comments to the Author** | **Comments of Authors** |
| --- | --- |
| **ACADEMIC EDITOR:**  The manuscript has improved a lot after the revision you submitted and the comments by the reviewers are quite encouraging. However, I still feel that with minor revision, your paper can gain more interest from the academic fraternity and provide valuable insights.  I suggest to incorporate the following: |  |
| - Mention the contribution of the study in the abstract and introduction sections. | **Thank you very much for your comments. This has been incorporated in the revised manuscript with track changes. (Line 41-44; 165-186)** |
| - Read carefully for the grammatical or punctuation errors ( I suggest getting help from the language expert) | **Thank you and well noted. This has been in cooperated in the revised manuscript with track changes.** |
| - Be consistent with reporting the results of the study as mentioned by one of the reviewers. | **Noted with thank you. This has been incorporated in the revised manuscript with track changes. (Line 524-529; 531-535; 540-550)** |
|  |  |
| **REVIEWERS:** 1. If the authors have adequately addressed your comments raised in a previous round of review and you feel that this manuscript is now acceptable for publication, you may indicate that here to bypass the “Comments to the Author” section, enter your conflict of interest statement in the “Confidential to Editor” section, and submit your "Accept" recommendation. |  |
| Reviewer #1: All comments have been addressed | **Thank you very much for valuable time spend with our research and provide us valuable comments.** |
| Reviewer #2: (No Response) | **Thank you very much for valuable time spend with our research and provide us valuable comments.** |
|  |  |
| 2. Is the manuscript technically sound, and do the data support the conclusions?  The manuscript must describe a technically sound piece of scientific research with data that supports the conclusions. Experiments must have been conducted rigorously, with appropriate controls, replication, and sample sizes. The conclusions must be drawn appropriately based on the data presented. |  |
| Reviewer #1: Yes | **Well, noted. Thank you for your comment.** |
| Reviewer #2: Partly | **Noted with thank you. This has been incorporated in the revised manuscript with track changes. (Line 524-529; 531-535; 540-550)** |
|  |  |
| 3. Has the statistical analysis been performed appropriately and rigorously? |  |
| Reviewer #1: Yes | **Well, noted. Thank you for your comment.** |
| Reviewer #2: Yes | **Well, noted. Thank you for your comment.** |
|  |  |
| 4. Have the authors made all data underlying the findings in their manuscript fully available?  The [PLOS Data policy](http://www.plosone.org/static/policies.action#sharing) requires authors to make all data underlying the findings described in their manuscript fully available without restriction, with rare exception (please refer to the Data Availability Statement in the manuscript PDF file). The data should be provided as part of the manuscript or its supporting information, or deposited to a public repository. For example, in addition to summary statistics, the data points behind means, medians and variance measures should be available. If there are restrictions on publicly sharing data—e.g. participant privacy or use of data from a third party—those must be specified. |  |
| Reviewer #1: Yes | **Well, noted. Thank you for your comment.** |
| Reviewer #2: Yes | **Well, noted. Thank you for your comment.** |
|  |  |
| 5. Is the manuscript presented in an intelligible fashion and written in standard English?  PLOS ONE does not copyedit accepted manuscripts, so the language in submitted articles must be clear, correct, and unambiguous. Any typographical or grammatical errors should be corrected at revision, so please note any specific errors here. |  |
| Reviewer #1: Yes | **Thank you very much for your comments.** |
| Reviewer #2: No | **Thank you and well noted. This has been in cooperated in the revised manuscript with track changes.** |
|  |  |
| 6. Review Comments to the Author  Please use the space provided to explain your answers to the questions above. You may also include additional comments for the author, including concerns about dual publication, research ethics, or publication ethics. (Please upload your review as an attachment if it exceeds 20,000 characters) |  |
| Reviewer #1: Authors have addressed all the comments and in my opinion manuscript has been reached to the publishing quality. And I believe that the paper has improved significantly. I found the language of the text to be pleasant and easy to follow. I have the manuscript, which has already been revised and refers to the comments of the previous reviewers. Therefore, I have only a few more comments that the authors could address: I would suggest the authors to emphasize more the scientific contribution of their research in the introduction. | **Thank you very much for your comments. This has been incorporated in the revised manuscript with track changes. (Line 41-44; 165-186)** |
| Reviewer #2: Please revise the grammatical and punctuational errors. Technically paper is rigorous. however, "Their Median Salary Level Q1 was Rs.25,000- 50,000 while in Q3, it was Rs.50,000- 70,000. Most employees (35.2%) have had working experience of between 5 to 9 years. Only 19 employees have worked for more than 25 years. In addition to that, 51 employees were with less than one year of experience. The descriptive statistics show the Median year in service Q1 was 5–9 years while Q3 was 1-4 years." Looks fuzzy to me. Please mention them in format 'median(Q1,Q3)'. | **Thank you very much for your comments. This has been incorporated in the revised manuscript with track changes. (Line 332-334; 338-339; 341-342)** |
|  |  |
| 7. PLOS authors have the option to publish the peer review history of their article ([what does this mean?](https://journals.plos.org/plosone/s/editorial-and-peer-review-process#loc-peer-review-history)). If published, this will include your full peer review and any attached files.   If you choose “no”, your identity will remain anonymous but your review may still be made public.   **Do you want your identity to be public for this peer review?** For information about this choice, including consent withdrawal, please see our [Privacy Policy](https://www.plos.org/privacy-policy). | **Yes. I would like to share peer review history.** |
| Reviewer #1: No |  |
| Reviewer #2: **Yes:** BIBHAV ADHIKARI |  |

Thank you very much for your valuable comments.

Sincerely,

Wasantha Rajapakshe

(Corresponding Author)
